# Apparent Amenorrhœa

**Published:** 1909-04-03

**Authors:** 


### Gynæcology.

### APPARENT AMENORRHŒA.

Cases in which menstruation occurs in a normal manner, but the menstrual fluid is unable to escape externally owing to closure of some part of the genital canal, are not of common occurrence. When, however, they do occur they are of great clinical interest from the point of view of treatment, especially when the lesion is congenital absence of the vagina, partial or complete. The great majority of the recorded cases are of congenital origin, most commonly due to an imperforate condition of the hymen; but absence of the whole or part of the vagina is fairly frequent, and closure of the cervix uteri has been described. The latter two lesions may be acquired, but it would seem that an imperforate hymen is always congenital. Closure of the vagina may occur as a result of a chronic granular vaginitis in infancy, the result of infection sometimes caused by the exanthems; scarlet fever is said to be the commonest source. It is, however, quite conceivable that infection from without other than that due to exanthems may cause a severe vaginitis with the formation of granulation tissue in the vagina and consequent complete fusion of the anterior and posterior vaginal walls. Acquired closure of the cervix is a very rare condition, but is said to have followed injury during labour, or cicatrisation after operations, such as amputation of the cervix. More commonly, however, trauma, whether accidental or intentional, leads only to stenosis of the cervical canal, not to complete closure. So also lacerations of the vagina lead to the formation of a mere fistulous track more commonly than complete closure. The suggestion that the constant flow of uterine secretions from above assists to prevent complete closure probably has much truth in it.

The clinical history of these cases is generally very clear, because the patients usually complain of the well-known menstrual molimina, which show that the function is established, and yet no secretion escapes. As a general rule advice is sought before the patient reaches the age of 20 in the congenital cases or those acquired during infancy. When the hymen is imperforate the condition is self-evident on inspection of the vulva, and combined recto-abdominal examination reveals the presence of a fluctuating swelling between the rectum and the bladder, with the uterus movable on its upper end.

There is one point, however, of great clinical importance which it is advisable to settle before treating the case, and that is to ascertain whether the uterus and the Fallopian tubes have also become distended with the menstrual secretions. The distension of the uterus along with the vagina is not of so much importance, but if a single or double hæmato-salpinx can be demonstrated it is absolutely essential that these should be dealt with before letting the retained fluid out of the vagina and uterus. The danger of overlooking such conditions is that a possible rupture of the tube or peritoneal leakage of its contents may occur whilst emptying the vagina. Cases are on record in which peritonitis has occurred from this accident. If a laparotomy is performed upon these cases the tubes may be emptied and their fimbriated extremities opened before emptying the vagina by a vulval incision. There would seem to be no adequate reason for removal of such dilated tubes unless it can be shown that they are hopelessly disorganised.

It is important clinically to adopt stringent antisepsis in opening a hæmato-colpos distended with menstrual fluid, for it must be remembered that any cavity which has never been exposed to the air is readily infected. It is also important to make no attempt to wash out the retained secretion, but simply to allow it to escape slowly into an aseptic dressing after putting in a large drainage tube to keep the incision open.

The treatment of absence of the vagina is a much more difficult matter, and the remarks concerning hæmato-salpinx apply to it also. There are two alternatives open: either completely to remove the distended uterus and, if affected, the Fallopian tubes by laparotomy, leaving the congenital vaginal absence alone; or to make an attempt to fashion a new vagina and drain the uterus through it after incising what appears to be its lower segment. The former alternative must be abhorrent, for it condemns the patient to a celibate life and much consequent mental suffering; on the other hand it is a difficult matter to make a vagina; but if the lower third or half only is absent it is well worth trying.

It is quite hopeless simply to dissect up between the rectum and bladder and to attempt to keep this track open by plugging. It always contracts into a mere fistulous track which is as useless as no vaginal canal at all. The only possible way of making a new vagina is to dissect up until the uterus is reached if the vagina is completely absent, or until the vagina is reached in partial atresia; and then after stopping all hæmorrhage to line the new canal with skin. It may be possible if only a short length of the canal is absent to draw down the vagina and attach it to the vulval incision, but this will be rarely feasible. As a general rule it will be necessary to form a skin lining either by taking flaps from the labia, or even the buttocks, and turning them up into the new canal; or else to make large skin grafts after Thiersch's method, and fix them securely in place by a few sutures. It will be rarely possible to get a good result from one operation, but if a skin flap or graft of any size can be got to remain in position its edges will grow and a second operation will then become the more hopeful. Great patience and ingenuity is required in performing any operation of this kind and the patient's confidence must be obtained. It is probably a good principle not to open the uterus to let out its contents until the new vagina is made. Very few operations of this nature have been performed, but the importance of the condition in an otherwise healthy individual makes it a matter of very serious consideration, before removal of the essential organs is carried out and the attempt to make a new vagina is abandoned.

